# Septic shock in pregnancy due to pyogenic sacroiliitis: a case report

**DOI:** 10.1186/1752-1947-3-6505

**Published:** 2009-03-13

**Authors:** María Lapresta Moros, Cesar Rodrigo, Adela Villacampa, Julián Ruiz, Carlos Lapresta

**Affiliations:** 1 Department of Obstetrics and Gynecology, Miguel Servet University Hospital, Zaragoza, Spain; 2 Department of Anaesthesia, Miguel Servet University Hospital, Zaragoza, Spain; 3 Department of Preventive Medicine, Miguel Servet University Hospital, Zaragoza, Spain

## Abstract

**Introduction:**

Lower back pain due to sacroiliac joint dysfunction is a common symptom during pregnancy. However, infection of the sacroiliac joint is rare, even more so if no predisposing factors are present.

**Case presentation:**

After the onset of unspecific acute pain in the left buttock region, a 31-year-old pregnant woman developed septic shock due to pyogenic sacroiliitis. The medical and obstetric management, treatment applied and patient's experience are described.

**Conclusion:**

The correct diagnosis and treatment of pyogenic sacroiliitis during pregnancy may avoid joint and bone destruction in addition to maternal and fetal complications.

## Introduction

The function of the sacroiliac joint is to reduce pelvic stress caused by changes in weight due to body movement. Hormonal effects of pregnancy permit relaxation of the ligaments supporting the sacrum and the pelvic bones. It has been hypothesized that pregnancy sacroiliitis is associated with microscopic areas of injury on the joint surfaces produced by the changes during pregnancy. Pyogenic sacroiliitis has also been related to immunosuppression during pregnancy.

Sacroiliac joint disease usually presents with lower back pain that increases with ambulation. The majority of cases represent non-specific and uncomplicated arthritis. Nevertheless, sometimes this joint can be seeded after bacteraemia, resulting in a pyogenic process. The prognosis depends on prompt diagnosis and early start of treatment.

## Case presentation

A 31-year-old woman, gravida-1 (23 weeks' gestation), para-0, presented with acute severe pain in her left buttock region radiating to the leg and increasing with ambulation. No underlying pathologies or drug abuse were present and no systemic symptoms were encountered. Backache was initially attributed to nerve compression. Nonsteroidal anti-inflammatory drugs and rest were prescribed.

After 4 days, the pain became worse. Physical examination revealed a temperature of 39.2 ºC, pulse rate of 111 beats/minute, respiratory rate of 43 breaths/minute and blood pressure of 100/50mmHg. The laboratory test results were significant for leukocytes of 5400/mm^3^ with left shift (92%), haematocrit of 24%, D-Dimer of 946.8μg/L and platelet count of 85,000/mm^3^. Chest X-ray showed images of bilateral pulmonary condensation.

The patient was admitted to the intensive care unit with a diagnosis of septic shock and acute respiratory distress. Doppler ultrasound examination of both legs and pulmonary arteriography disproved the diagnosis of pulmonary embolism. An echocardiogram did not find any evidence of endocarditis.

Treatment with broad-spectrum antibiotics (gentamicin and ceftriaxone), inotropic drugs and ventilatory support was prescribed. Her general status improved throughout the following days. Nevertheless, her back pain became worse. A magnetic resonance imaging (MRI) scan revealed left-sided sacroiliitis with a small abscess at the lower joint margin extending into the iliac notch. A computed tomography-guided aspiration of the abscess was performed and the patient reported partial relief of her symptoms.

Sacroiliac aspiration yielded a small amount of fluid. Although blood cultures were positive for *Staphylococcus aureus*, culture of the material from the sacroiliac aspiration failed to yield positive results. Intravenous cloxacillin was added to the antibiotic therapy and a rehabilitation programme was initiated so that the patient might recover her strength and mobility.

**Figure 1:**
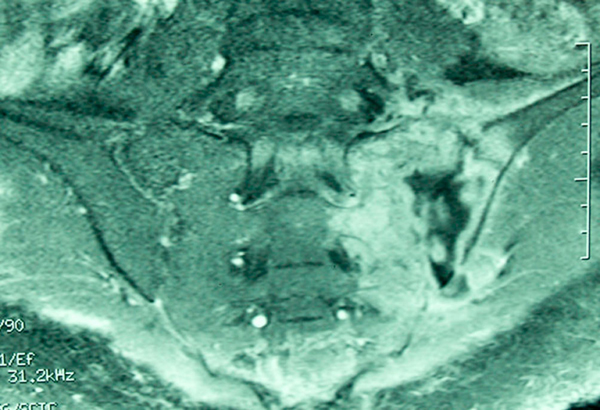
**Magnetic resonance imaging scan of the pelvis showing the left sacroiliac joint abscesses with sacroiliac joint destruction and focal osteomyelitis**.

A new MRI performed 6 weeks later showed progression of sacroiliac joint destruction and focal osteomyelitis (Figure 1). A cesarean section was performed under general anaesthesia at 34 weeks' gestation and a 2570g male neonate was delivered.

**Figure 2:**
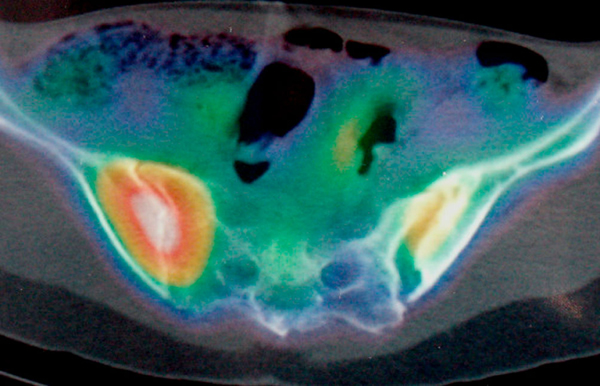
**Single-photon emission computed tomography/computed tomography demonstrating a widened left sacroiliac joint with active bone remodelling and moderate inflammatory activity**.

The total length of intravenous antibiotic treatment was 8 weeks. She continued with oral rifampicin and ofloxacin for 4 months. After cesarean section, the patient noticed progressive decreased pain and increased ambulatory ability. One year after the onset of symptoms, Technetium-99 conjugated with methylene diphosphonate (Tc-99m MDP) bone scintigraphy still demonstrated increased uptake in the left sacroiliac region. Figure 2 shows single-photon emission computed tomography/computed tomography (SPECT/CT) results. The patient is doing well with normal ambulation, although she continues to experience mild discomfort in her left buttock.

## Discussion

Sacroiliac joint infection is considered uncommon and usually related to trauma, illicit drug addiction or underlying diseases. In our patient, there was an unremarkable past medical history and she denied a history of trauma or drug abuse.

The presence of acute pyogenic sacroiliitis without predisposing conditions and the non-specific clinical presentation may delay diagnosis, especially when considering that lower back pain is a common symptom in pregnancy and postpartum. The diagnosis of pyogenic sacroiliitis during pregnancy requires a degree of clinical suspicion and should be confirmed by imaging diagnostic methods. Plain radiography may give normal images in early disease. There may be blurring of joint margins, a widened joint space or periarticular erosion. Radioisotopic bone scans have high specificity and sensibility for localizing bone inflammation but should not be used during pregnancy. Nonetheless, bone scans are helpful to check treatment response during the postpartum period.

MRI is probably the imaging diagnosis method of choice in pregnancy to detect pyogenic sacroiliitis. It provides a detailed evaluation of the joint and surrounding soft tissue without exposing the fetus to ionizing radiation.

Vaginal delivery could have been attempted in our patient. Epidural analgesia was considered to be contraindicated because of the risk of a disseminated infection to the spinal cord and meninges. Local or general anaesthesia might be other alternatives to relieve pain. As the patient was suffering severely from pain, the decision to perform a caesarean section was made on the basis of avoiding pain and joint distraction during delivery. There is no consensus on the appropriate way for delivery of patients with active pyogenic sacroiliitis [6].

*Staphylococcus aureus* is the most common cause of infectious sacroiliitis. Other conditions such as brucellosis or tuberculosis may produce sacroiliitis [7]. However, in both entities, clinical course is chronic. Embolic septic events in the setting of bacterial endocarditis may also be responsible for infectious sacroiliitis [8]. All of these conditions were excluded in our patient.

## Conclusion

Septic sacroiliitis, although uncommon, should be considered in pregnant patients who present with acute severe localized pain and fever, even when no predisposing factors are discovered. Delay in recognition and lack of therapy may result in severe complications. Pyogenic sacroiliitis complications include not only joint and bone destruction, but also maternal and neonatal septicaemia. Prompt diagnosis and treatment may avoid life-threatening complications for the mother and fetus.

## Consent

Written informed consent was obtained from the patient for publication of this case report and any accompanying images. A copy of the written consent is available for review by the Editor-in-Chief of this journal.

## Competing interests

The authors declare that they have no competing interests.

## Authors' contributions

ML wrote the case report and conducted the literature search. CR prepared the figures. AV and JR were involved in conception of the article. CL critically revised the manuscript. All authors were involved with treatment of this patient and all read and approved the final manuscript.
